# Methane prediction equations including genera of rumen bacteria as predictor variables improve prediction accuracy

**DOI:** 10.1038/s41598-023-48449-y

**Published:** 2023-12-02

**Authors:** Boyang Zhang, Shili Lin, Luis Moraes, Jeffrey Firkins, Alexander N. Hristov, Ermias Kebreab, Peter H. Janssen, André Bannink, Alireza R. Bayat, Les A. Crompton, Jan Dijkstra, Maguy A. Eugène, Michael Kreuzer, Mark McGee, Christopher K. Reynolds, Angela Schwarm, David R. Yáñez-Ruiz, Zhongtang Yu

**Affiliations:** 1 Department of Animal Sciences, The Ohio State University, Columbus, OH 43210 USA; 2 Department of Statistics, The Ohio State University, 2029 Fyffe Road, Columbus, OH 43210 USA; 3 Department of Animal Science, The Pennsylvania State University, University Park, PA USA; 4 Department of Animal Science, University of California, Davis, CA USA; 5 AgResearch Limited, Grasslands Research Centre, Palmerston North, 4442 New Zealand; 6 Wageningen Livestock Research, Wageningen University & Research, Wageningen, The Netherlands; 7 Milk Production, Production Systems, Natural Resources Institute Finland (Luke), 31600 Jokioinen, Finland; 8 School of Agriculture, Policy, and Development, University of Reading, Reading, UK; 9 Animal Nutrition Group, Wageningen University & Research, Wageningen, The Netherlands; 10 INRAE UMR Herbivores, VetAgro Sup, Université Clermont Auvergne, Saint-Genès-Champanelle, France; 11 Institute of Agricultural Sciences, ETH Zurich, Zurich, Switzerland; 12 Teagasc, AGRIC, Grange, Dunsany., CO., Meath, Ireland; 13 Department of Animal and Aquacultural Sciences, Norwegian University of Life Sciences, Ås, Norway; 14 Estación Experimental del Zaidin (CSIC), Granada, Spain; 15 Present Address: Consultoria, Piracicaba, SP Brazil

**Keywords:** Microbiology, Microbial communities, Environmental microbiology

## Abstract

Methane (CH_4_) emissions from ruminants are of a significant environmental concern, necessitating accurate prediction for emission inventories. Existing models rely solely on dietary and host animal-related data, ignoring the predicting power of rumen microbiota, the source of CH_4_. To address this limitation, we developed novel CH_4_ prediction models incorporating rumen microbes as predictors, alongside animal- and feed-related predictors using four statistical/machine learning (ML) methods. These include random forest combined with boosting (RF-B), least absolute shrinkage and selection operator (LASSO), generalized linear mixed model with LASSO (glmmLasso), and smoothly clipped absolute deviation (SCAD) implemented on linear mixed models. With a sheep dataset (218 observations) of both animal data and rumen microbiota data (relative sequence abundance of 330 genera of rumen bacteria, archaea, protozoa, and fungi), we developed linear mixed models to predict CH_4_ production (g CH_4_/animal·d, ANIM-B models) and CH_4_ yield (g CH_4_/kg of dry matter intake, DMI-B models). We also developed models solely based on animal-related data. Prediction performance was evaluated 200 times with random data splits, while fitting performance was assessed without data splitting. The inclusion of microbial predictors improved the models, as indicated by decreased root mean square prediction error (RMSPE) and mean absolute error (MAE), and increased Lin’s concordance correlation coefficient (CCC). Both glmmLasso and SCAD reduced the Akaike information criterion (AIC) and Bayesian information criterion (BIC) for both the ANIM-B and the DMI-B models, while the other two ML methods had mixed outcomes. By balancing prediction performance and fitting performance, we obtained one ANIM-B model (containing 10 genera of bacteria and 3 animal data) fitted using glmmLasso and one DMI-B model (5 genera of bacteria and 1 animal datum) fitted using SCAD. This study highlights the importance of incorporating rumen microbiota data in CH_4_ prediction models to enhance accuracy and robustness. Additionally, ML methods facilitate the selection of microbial predictors from high-dimensional metataxonomic data of the rumen microbiota without overfitting. Moreover, the identified microbial predictors can serve as biomarkers of CH_4_ emissions from sheep, providing valuable insights for future research and mitigation strategies.

## Introduction

As a potent greenhouse gas that contributes significantly to climate change, methane (CH_4_) emissions from ruminants pose a direct threat to the environment and sustainable agricultural production^1^. Methane emissions from ruminants also lead to a waste of part of the ingested feed energy^2^. In sheep, CH_4_ emissions result in approximately a 7% loss of gross energy intake^3^. In recent years, there has been growing interest in prediction models for CH_4_ emissions for several reasons. First, direct measurement of CH_4_ emissions from large herds or flocks of ruminants is technically difficult and time-consuming, and thus it can be done practically only with small numbers of animals in research settings^4^. Second, it is not practical or feasible to establish regional, national, or global inventories of CH_4_ emissions by direct measurement of CH_4_ emissions from individual animals. Third, statistical models can assist in describing and understanding the relationship among CH_4_ emissions, animal performance, and other factors (nutritional, microbial, and physiological).

A diverse set of prediction models has been developed to predict enteric CH_4_ emissions from sheep^5^ and cattle^6,7^. These prediction equations include predictor variables describing the dietary composition, feed intake, rumen fermentation profiles, and other animal traits such as body weight (BW), but not individual taxa (genera or species) of the rumen microbiota. These prediction models vary considerably in robustness and prediction accuracy^8,9^. The rumen microbiota is solely responsible for CH_4_ emissions from ruminants. Specifically, rumen bacteria, fungi, and protozoa digest and ferment the ingested feed and produce the substrates (methylamines, hydrogen, CO_2_, and formate) that are then used by the rumen methanogens to produce CH_4_^10^. Thus, the rumen microbiota is directly responsible for the CH_4_ emitted by ruminants although it can be affected by several factors, including diet and host-related factors^11^. Indeed, several studies have reported strong associations or correlations of some bacteria, methanogens, or protozoa with or qualitatively indicative of CH_4_ production^12,13^. In this context, we hypothesized that the inclusion of individual taxa of the ruminal microbiota as predictor variables, in addition to animal and feed-related predictor variables, in CH_4_ prediction models would improve our ability to predict CH_4_ emissions. Methane prediction equations or models containing rumen microbial taxa as predictor variables may also help understand their roles and importance in enteric CH_4_ emissions. The objective of this study was to develop such prediction models using multiple statistical/machine learning (ML) methods and a dataset that contained both animal-related data and metataxonomic data of the rumen microbiota of a cohort of sheep that were not subjected to any dietary interventions.

## Methods

### Dataset

This study did not involve the use of any animals, and thus no approval of the Institutional Animal Care and Use Committee was required. The dataset (Supplementary Table 1) used in this study was generated in a previous study that compared the rumen microbiota and CH_4_ emissions among a group of New Zealand sheep that were not subjected to any dietary interventions^13^. The animal experiment and analyses were detailed in another report^14^. Briefly, 1225 sheep were fed a pelleted alfalfa diet (without any anti-methane inhibitors) twice daily at two times their maintenance requirement for energy (based on BW). CH_4_ emissions from 340 individual animals were measured with open-circuit respiration chambers twice in two independent measuring rounds (10–15 days apart) with each time lasting for two consecutive days^14^. CH_4_ production (g CH_4_/animal·d) and CH_4_ yield (g CH_4_/kg of dry matter intake, DMI) were calculated. Feed intake and BW of the 340 individual animals were also recorded. Rumen samples were collected from 118 of the 340 sheep, with 60 representing the highest and 58 representing the lowest CH_4_ yield sheep, immediately after each CH_4_ emission measurement (corresponding to 17–18 h after the last feeding) using the stomach tubing method. The two groups of sheep were balanced by BW (24–60 kg), sex, and breeds (indicated below). The rumen fermentation characteristics, including acetate:propionate (A:P) ratio, were analyzed using gas–liquid chromatography. We provided the density plots of the CH_4_ data (g/d and g/kg of DMI, Supplementary Fig. 1). Both density curves appeared to represent the mixture of the two groups, which is consistent with the data feature (high and low emission sheep) and with the patterns found in the previous study^13^.

Two rumen samples were collected (10 to 15 days apart) from each sheep and the composition and structure of the rumen microbiota were analyzed using metataxonomics sequencing the 16S rRNA genes of both bacteria and archaea, 18S rRNA gene of protozoa, and internal transcribed spacer 1 (ITS1) of fungi^13^. One sheep was excluded due to missing data, and 16 sheep were represented by only one sample that yielded complete metataxonomic data. This resulted in a cumulative 218 observations, with 101 sheep each contributing two observations and the remaining 16 sheep contributing one observation.

In this study, we retrieved the metataxonomic sequencing data of the sheep rumen samples from the EMBL database per the accession numbers (ERP003779 for Bacteria, ERP003773 for Archaea, ERP003772 for ciliate protozoa, and ERP003764 for anaerobic fungi). The sequences were then analyzed using DADA2^15^. Briefly, the amplicon sequences were clustered into amplicon sequence variants (ASVs), which were then taxonomically assigned to individual taxa using specialty databases: the Silva database (version 138.1) for bacteria, archaea, and protozoa^16^; and the UNITE database for fungi^17^. The relative sequence abundance of each genus was calculated as the sum of the relative sequence abundance of all the ASVs assigned to the respective genus. The sequences of the ASVs that were classified to an unclassified genus (Uncl_genus) are listed in Supplementary Table 2. Only the genera that each had a relative sequence abundance reaching > 1% in all the samples were used as microbial variables. For an Uncl_genus, its higher and known taxon name was used to show its taxonomic lineage. In total, 330 microbial genera were obtained and used, including 308 genera of bacteria, 9 genera of archaea, 5 genera of protozoa, and 8 genera of fungi. The relative sequence abundance data of the microbial genera were log-transformed before the modeling process. In addition to the rumen microbiota data and CH_4_ emission data (CH_4_ production, g CH_4_/animal·d; CH_4_ yield, g CH_4_/kg of DMI), the dataset also contained the following five animal-related data of the individual sheep: DMI (kg/d), BW (kg), A:P ratio, sex, and breed (Coopworth, Corriedale, Finnish Landrace, Perendale, Romney, and Texel).

### Linear mixed model

In our dataset, most animals (101 out of 118) had two records of CH_4_ emissions and other data (except for sex and breed). These repeated measurements from the same sheep were likely to be more akin to each other than to the measurements from a different sheep. Thus, the two records from the same sheep were expected to be correlated. Linear mixed-effects models were used to model the dependence of the data and account for correlations between the two repeated measurements. The general structure of the model was described as follows:

1 $${y}_{ij}={{\varvec{X}}}_{{\varvec{i}}}{\varvec{\beta}}+{\alpha }_{i}+{\varepsilon }_{ij}$$

where y_*ij*_ is the observed CH_4_ emissions from the *i*th animal and *j*th observation (*i* = 1, …, 118 and *j* = 1, 2), set as either g CH_4_/animal·d (referred to as ANIM-B models) or g CH_4_/kg of DMI (referred to as DMI-B models); **X**_*i*_ = (1, DMI, BW, A:P ratio, sex, breed, microbial variables) is the design vector for the fixed effects containing the values of the predictor variables for the *i*th animal and the “1” entry for the intercept (DMI was not included as a variable in developing the DMI-B models because it is a dependent variable in those models); **β** is the coefficients vector of the fixed effects describing the relationships between CH_4_ emissions and the predictor variables; *α*_*i*_ is the random animal effects to account for intra-animal correlation between the two measurements; and ε_*ij*_ is the random error. The *α*_*i*_ was assumed to be independent and identically distributed with mean zero and variance ${{\sigma }_{\alpha }}^{2}$, and the ε_*ij*_ errors were assumed to be independent and identically distributed with mean zero and variance ${\sigma }^{2}$. Further, it was assumed that random effects and errors were mutually independent.

### Machine learning methods

The rumen microbiota is diverse, containing many hundreds to thousands of distinct species of microbes at varying relative sequence abundance. The inclusion of such a large number of microbial variables in prediction models can lead to overfitting. Additionally, relative abundance data of microbiota are usually on different scales, zero-inflated, sparse, and over-dispersed. Thus, proper selection of microbial predictor variables to be included in prediction models is crucial in model development. In the present study, we used four ML methods to select microbial predictor variables: random forest combined with boosting (RF-B) using the randomForest and gbm packages^18^, least absolute shrinkage and selection operator (LASSO) using the glmnet package^19^, generalized linear mixed model with LASSO (glmmLasso) using the glmmLasso package^20^, and smoothly clipped absolute deviation (SCAD) using the splmm package^21^. We opted for both LASSO and glmmLasso because the former does not consider intra-animal correlation, while the latter takes these correlations into account by utilizing the linear mixed-effect model described in Eq. (1). Each of the ML methods was evaluated for both prediction performance and fitting performance. Specifically, to evaluate prediction performance (based on 200 evaluations with random data splitting into testing data and training data (see section ‘Evaluation of prediction performance’ below), each ML method was used to make variable selection (selection for both animal and microbial predictor variables). Using the lmer function of the lme4 package^22^, we also fitted conventional models utilizing solely the animal-related data (i.e., BW, A:P ratio, and DMI (DMI was used only in developing the ANIM-B models)) as predictors. The two categorical variables (sex and breed) were not included in the prediction models because their addition did not lead to improvement in prediction performance (data not shown). The sets of variables selected by glmmLasso, LASSO, SCAD, RF-B, and conventional modeling were then separately fitted with a linear mixed model, and the resultant models were used to predict CH_4_ emissions. Further, to evaluate fitting performances, linear mixed models were fitted with all available data by selecting the variables using the ML and the conventional method. The Akaike information criterion (AIC) and Bayesian information criterion (BIC) of those models were computed using the ANOVA function in R v4.0.2^23^. The estimated standard deviation of the animal random effects (${\widehat{\sigma }}_{\alpha }$), coefficients, and associated standard errors (SE) of the predictor variables were computed using the lmer function^22^. The observations and residuals versus the model predictions were plotted to evaluate the linear mixed model assumptions for all the ML models.

### Evaluation of prediction performance

Prediction performance was validated in five steps. Step 1 involved screening the independent variables using the sure independence screening (SIS) test implemented in the SIS package in R^24^. This step was omitted for the RF-B method because this method can handle high-dimensional data without the need for data prescreening when building the decision trees. To check for potential multicollinearity, the variance inflation factor (VIF) was examined, and variables with a VIF < 5 were retained as recommended by Niu et al.^7^.

Step 2 began by randomly splitting the dataset into testing data (20% of the data) and training data (the remaining data). The training data were standardized by mean centering (subtracting each variable from the corresponding mean) and scaling (dividing each variable by its corresponding standard deviation). The testing data were standardized based on the centering and scaling from the training data. To prevent data leaking of the repeated records from the same animal, the two repeated records were always allocated to either the testing data or the training data.

In step 3, glmmLasso, LASSO, SCAD, and RF-B were individually used to select predictor variables from the training data. For glmmLasso and SCAD, the optimal tuning parameter *λ* was determined using BIC. Two increments of *λ*, 0.1 and 0.5, were also evaluated, but the results were virtually the same (data not shown) except for a substantial increase in computing time. For LASSO, tenfold cross-validation was used to determine the optimal tuning parameter *λ*. For RF-B, the top 10 important variables identified using the randomForest package were selected when the number of decision trees was set as 500, and the number of variables randomly sampled as candidates at each split (using mtry in the randomForest package) was set as 40. The top 10 important variables determined by boosting using the gbm package were also selected with the default settings applied. The union of the variables selected by random forest and boosting was kept.

Step 4 involved separately refitting a linear mixed model with the sets of selected variables using each of the ML methods and the training data. Predictions were then made using the values of the variables from the testing data. All the predictions and observations were recorded.

Step 5 repeated steps 2 to 4 for 200 times (sufficiently large and not too computationally intensive) by re-splitting the data into testing and training data in each iteration. Mean squared prediction error (MSPE), root MSPE (RMSPE), mean absolute errors (MAE), and Lin’s concordance correlation coefficients (CCC) were calculated using the equations specified in the section ‘Model evaluation equations’ below. We utilized RMSPE, MAE, and CCC to provide a comprehensive evaluation of the model performance. In particular, MAE is a robust assessment that is less sensitive to extreme values compared to RMSPE, whereas CCC can provide information on the precision and accuracy of the prediction.

### Model evaluation equations

The values of MSPE, RMSPE, and MAE of all the models were calculated using the equations shown below^25^. The unit of MSPE was (*g/d*)^2^ for the ANIM-B models and (*g/kg*)^2^ for the DMI-B models. Lin’s CCC was calculated by the epi.ccc function in the epiR package in R^26^.

$$MSPE=\frac{\sum_{i=1}^{N}{{(O}_{i}-{P}_{i})}^{2}}{N}$$

$$MAE=\frac{{\sum }_{i=1}^{N}\left|{(O}_{i}-{P}_{i})\right|}{N}$$

$$RMSPE= \sqrt{MSPE}$$

$$CCC=\frac{2{s}_{op}}{{s}_{o}^{2} + {s}_{p}^{2}+{(\overline{o  }-\overline{p })}^{2}}$$

where ${O}_{i}$ is the CH_4_ emissions of *i*th observation; ${P}_{i}$ is CH_4_ prediction from the model; N is the total number of observations; ${S}_{o}$ is the standard deviation of the observations; ${S}_{p}$ is the standard deviation of the predictions; $\overline{o }$ is the mean of the observations; $\overline{p }$ is the mean of the predictions; ${S}_{op}$ is the covariance of the observations and the predictions.

The R codes we used in the present study were available at GitHub (https://github.com/yu2269/CH_4__prediction_model).

## Results

### Model performance

Tables 1 and 2 summarize the prediction performances of the ANIM-B and the DMI-B models developed using both the ML and the conventional methods, based on random data splitting (200 times). The ML methods notably improved the performance of ANIM-B models, reducing RMSPE (by − 1.25% for SCAD and 3.8% for glmmLasso) and MAE (by − 0.39% for SCAD and 5.08% for LASSO), while increasing CCC (by 2.64% for SCAD and 9.8% for LASSO), as compared to the conventional method. Similarly, the ML methods enhanced the performance of DMI-B models, reducing RMSPE (by 6.22% for glmmLasso and 11.12% for LASSO) and MAE (by 7.36% for RF-B and 13.31% for LASSO) and increasing CCC (by 68.73% for glmmLasso and 106.18% for LASSO). Tables 3 and 4 present the fitting performance of the ANIM-B and the DMI-B models developed using both the ML methods and the conventional method, based on the entire dataset. Compared to the conventional method, the ML methods decreased BIC (by − 33 for LASSO and 19 for glmmLasso) and AIC (by − 152 for LASSO and 53 for glmmLasso) for the ANIM-B models. Similarly, for the DMI-B models, the ML methods decreased BIC (by − 201 for RF-B and 5 for glmmLasso and SCAD) and AIC (by − 157 for RF-B and 18 for glmmLasso), as compared to the conventional method.

**Table 1:** Prediction performance of the animal-based (ANIM-B) models (CH_4_ production; g/d) developed using conventional method and four machine learning methods.

	Conventional	glmmLasso	LASSO	SCAD	RF-B
RMSPE	2.96	2.85	2.85	3.00	2.91
Reduction of RMSPE (%)	–	3.80	3.62	-1.25	1.52
MAE	2.29	2.18	2.17	2.29	2.21
Reduction of MAE (%)	–	4.60	5.08	-0.39	3.50
CCC	0.64	0.70	0.71	0.66	0.68
Increase of CCC (%)	–	9.49	9.80	2.64	5.29

*The conventional method only used animal-related data; the relative abundance of all the microbial data was log-transformed; glmmLasso, generalized linear mixed model combined with LASSO; LASSO, least absolute shrinkage and selection operator; SCAD, smoothly clipped absolute deviation implemented on linear mixed models; RF-B, random forest combined with boosting. The data were randomly split into a training set and a testing set (80:20) 200 times and were standardized by mean centering and scaling (detailed in “Methods”).

**Table 2:** Prediction performance of the dry matter intake-based (DMI-B) models (CH_4_ yield; g/kg DMI) developed using conventional method and four machine learning methods.

	Conventional	glmmLasso	LASSO	SCAD	RF-B
RMSPE	2.12	1.99	1.89	1.92	1.99
Reduction of RMSPE (%)	–	6.22	11.12	9.66	6.50
MAE	1.65	1.51	1.43	1.48	1.52
Reduction of MAE (%)	–	8.21	13.31	9.97	7.36
CCC	0.28	0.46	0.57	0.51	0.48
Increase of CCC (%)	–	68.73	106.18	84.36	73.09

See the notes of Table 1.

**Table 3:** Fitting performance of the animal-based (ANIM-B) models (CH_4_ production; g/d) fitted with the variables selected from all the available data by the conventional method and machine learning methods.

	Conventional	glmmLasso	LASSO	SCAD	RF-B
BIC	1070	1051	1103	1065	1095
Reduction of BIC	–	19	− 33	5	− 25
AIC	1050	997	1202	1028	1031
Reduction of AIC	–	53	− 152	22	19
Number of variables	3	13	27	8	17

See the notes of Table 1.

**Table 4:** Fitting performance of the dry matter intake-based (DMI-B) models (CH_4_ yield; g/kg DMI) fitted with the variables selected from all the available data by the conventional method and machine learning methods.

	Conventional	glmmLasso	LASSO	SCAD	RF-B
BIC	894	889	969	889	1095
Reduction of BIC	–	5	− 75	5	− 201
AIC	874	856	868	859	1031
Reduction of AIC	–	18	6	15	− 157
Number of variables	2	9	25	6	17

See the notes of Table 1.

The final models were selected by balancing the prediction performance and the fitting performance. Among the ANIM-B models, the glmmLasso model had the lowest BIC and AIC (Table 3). It also had the lowest RMSPE in prediction performance (Table 1). Thus, we selected the ANIM-B glmmLasso model as the final model. For the prediction performance of the DMI-B models, the LASSO model had a lower RMSPE than the SCAD model (1.89 vs. 1.92), which was lower than that of the glmmLasso model (Table 2), but the LASSO model had a higher BIC and AIC than the SCAD model and the glmmLasso model (Table 4). The DMI-B SCAD model also had the fewest variables (6 in total, Table 4) among the ML models. Thus, the DMI-B SCAD model was selected as the final DMI-B model.

We assessed the performance of the models by plotting the observed CH_4_ emissions against the CH_4_ emissions predicted using the final ANIM-B and the DMI-B models, along with the corresponding studentized residuals (Supplementary Figs. 2 and 3, respectively). Both the ANIM-B glmmLasso and the DMI-B SCAD models showed more tightly clustered and less scattered patterns in the observation vs. prediction plots, as compared to the ANIM-B and the DMI-B conventional models (Supplementary Figs. 2A,B and 3A,B, respectively). On the studentized residuals vs. predictions plots, the two ANIM-B and the DMI-B ML models also had tighter and more symmetric patterns around the regression lines that had smaller slopes compared to the ANIM-B and the DMI-B conventional models (Supplementary Figs. 2a,b and 3a,b, respectively).

### Animal-based (g CH_4_/animal·d) prediction models

The final ANIM-B glmmLasso model contained three animal-related predictor variables (DMI, A:P ratio, and BW) and 10 bacterial genera (or equivalents) of rumen bacteria out of the 308 genera (Table 5). None of the protozoal, fungal, or archaeal genera was selected as predictor variables. The three animal-related variables all had a positive coefficient. Of the 10 microbial predictor variables, four had a positive coefficient: 0.59 for the Uncl_genus of *Oscillospiraceae*, 0.49 for the Uncl_genus of the order *Clostridia*, 0.46 for the Uncl_Family of the order *Gastranaerophilales*, and 0.24 for the genus *Moryella*. The remaining six genera had a negative coefficient: Uncl_Family of the order *RF39* (‒0.27), the genus *Prevotella_7* (‒0.30), the Uncl_Genus of *Marinifilaceae* (‒0.34), the genus *Syntrophococcus* (‒0.37), the genus *Oribacterium* (‒0.40), and the Uncl_Family of the order *Oscillospirales* (‒0.44). The ANIM-B conventional model had the same three of the five animal-related data as predictor variables (DMI, A:P ratio, and BW). Compared to the ANIM-B conventional model, the ANIM-B glmmLasso model had a reduced ${\widehat{\sigma }}_{\alpha }$ (1.59 vs. 2.04), RMSPE (by 3.8%), and MAE (by 4.6%), and increased CCC (by 9.49%). The ANIM-B glmmLasso model also had a lower AIC and BIC than the ANIM-B conventional model (by 53 and 19, respectively).

**Table 5:** Coefficients and the associated standard errors (SE) of the predictor variables of the final ANIM-B (CH_4_ production; g/d) model.

	Predictor variables	glmmLasso^1^	Conventional^2^
Animal	Intercept	21.9 (0.19)	21.9 (0.23)
	Body weight (kg)	1.17 (0.24)	1.48 (0.18)
	Dry matter intake (kg/d)	1.35 (0.24)	1.18(0.25)
	Acetate to propionate (A:P) ratio	1.17 (0.18)	1.29 (0.26)
Bacteria**	Uncl_Genus of *Oscillospiraceae*	0.59 (0.17)	
	Uncl_Genus of the order *Clostridia*	0.49 (0.16)	
	**Uncl_Family of the order *Gastranaerophilales***	0.46 (0.17)	
	Genus *Moryella*	0.24 (0.15)	
	Uncl_Family of the order *RF39*	− 0.27 (0.16)	
	Genus *Prevotella 7*	− 0.30 (0.18)	
	Uncl_Genus of *Marinifilaceae*	− 0.34 (0.16)	
	Genus *Syntrophococcus*	− 0.37 (0.17)	
	**Genus *Oribacterium***	− 0.40 (0.17)	
	Uncl_Family of *Oscillospirales*	− 0.44 (0.18)	
Number of predictors		13	3
$\hat{\sigma }_{\alpha }$		1.59	2.04

The bolded genera were shared between the ANIM-B and the DMI-B models.

*Uncl* unclassified.

For all the models, the predictor variables were centered and scaled to have a mean of 0 and variance of 1.

**Log-transformed relative sequence abundance.

^1^Developed using glmmLasso and all the available data after log transformation of the microbial data.

^2^Developed using conventional model and only animal-related data.

### ***Dry matter intake-based (g CH***_***4***_***/kg of DMI) prediction model***

The final DMI-B SCAD model had six predictor variables: one animal-related predictor variable (A:P ratio) and five bacterial genera as microbial predictor variables (Table 6). No protozoal, fungal, or archaeal genera were selected as predictor variables. The A:P ratio predictor variable had a positive coefficient. Except for Uncl_Family of the order *Gastranaerophilales* (with a coefficient of 0.50), the other four microbial predictor variables were all known genera, all of which had a negative coefficient: − 0.24 for *Pseudoramibacter*, − 0.33 for *Megasphaera*, − 0.39 for *Selenomonas*, and − 0.40 for *Oribacterium*. The DMI-B conventional model had two animal-related predictor variables (A:P ratio and BW), and both had a positive coefficient. Compared to the DMI-B conventional model, the DMI-B SCAD model had a reduced ${\widehat{\sigma }}_{\alpha }$ (0.98 vs. 1.47), RMSPE (by 9.66%), and MAE (by 9.97%), and increased CCC (by 84.36%). Compared to the DMI-B conventional model, the DMI-B SCAD model reduced both AIC and BIC (by 15 and 5, respectively).

**Table 6:** Coefficients and the associated standard errors (SE) of the predictor variables of the final DMI-B (CH_4_ yield; g/kg DMI) model.

	Predictor variables	SCAD^1^	Conventional^2^
Animal	Intercept	15.13 (0.13)	15.12 (0.17)
	Body weight (kg)	–	0.89 (0.13)
	Acetate to propionate (A:P) ratio	0.93 (0.12)	0.25 (0.16)
Bacteria**	**Uncl_Family of the order *Gastranaerophilales***	0.50 (0.12)	
	Genus *Pseudoramibacter*	− 0.24 (0.12)	
	Genus *Megasphaera*	− 0.33 (0.12)	
	Genus *Selenomonas*	− 0.39(0.13)	
	**Genus *Oribacterium***	− 0.40 (0.12)	
Number of predictors		6	2
$\hat{\sigma }_{\alpha }$		0.98	1.47

The bolded genera were shared between the ANIM-B and the DMI-B models.

*Uncl* unclassified.

For all models, the predictor variables were centered and scaled to have a mean of 0 and variance of 1.

**Log-transformed relative sequence abundance.

^1^Developed using SCAD and all the available data after log-transformation of the microbial data.

^2^Developed using conventional model and only animal-related data except DMI.

## Discussion

The rumen microbiota is responsible for about 90% of the CH_4_ emitted from sheep^27^. With respect to CH_4_ production, rumen microbes can be categorized as producers of methanogenesis substrates (primarily bacteria and protozoa), CH_4_ producers (i.e., methanogens), and those that influence CH_4_ production by interacting with the above two categories either positively (e.g., through mutualism and commensalism) or negatively (i.e., through amensalism, competition, and predation) interactions. Thus, some of the rumen microbes could have a quantitative relationship with the overall CH_4_ emissions. Using four statistical/ML methods and one dataset (218 observations) that contains both animal-related data and metataxonomic data of the rumen microbiota (330 genera in total), we developed ML models for improved prediction of CH_4_ production (g CH_4_/animal·d) and CH_4_ yield (g CH_4/_kg of DMI) from sheep, which include rumen microbes as predictor variables.

### New ML models including microbial predictor variables

As evaluated for prediction performance, it is evident that all the ML models, except for the ANIM-B SCAD model, improved prediction of CH_4_ emissions in terms of error, bias, and accuracy when compared with the conventional models. Additionally, the improvement in prediction performance demonstrated in the evaluation with testing data randomly selected from the entire dataset suggests the potential usefulness of these models over a broad range of CH_4_ emissions. The improvement in CH_4_ prediction by the final ANIM-B glmmLasso model and the final DMI-B SCAD model is also evident in the plots of the predictions vs. the observations along with the studentized residuals. In addition to the same animal-related predictor variables included in the conventional models (except for the exclusion of BW in the DMI-B SCAD model), the final ML included genera of bacteria as predictor variables. The improvement in prediction performance strongly suggests that the inclusion of these microbial predictor variables might have fine-tuned the models. Moreover, the improved predictions of the final ML models align well with the assumptions of linear mixed models. These findings suggest that microbial predictor variables can differentiate sheep based on CH_4_ production levels, offering promising prospects for capturing variations in CH_4_ emissions and helping achieve accurate CH_4_ prediction.

### The bacterial genera included in the ML models

Despite the dataset containing 330 genera of microbes, only a small number of genera were selected as microbial predictor variables, and their inclusion did not lead to model overfitting. Notably, all the microbial predictor variables in both the ANIM-B and the DMI-B prediction models are genera of bacteria, with the majority belonging to *Firmicutes*, an abundant phylum of rumen microbiota. Interestingly, no archaea, protozoa, or fungi were selected as predictor variables in any of the ML models. The absence of methanogen genera in the models might seem counterintuitive, given their role as CH_4_ producers. However, previous studies have shown that the abundance of rumen methanogens, even that of the most abundant genus, *Methanobrevibacter*^28^, has very a weak to no correlation with CH_4_ production^12,29^. Furthermore, changes in diets that significantly affected CH_4_ production were not found to lead to substantial alterations in the relative sequence abundance of *Methanobrevibacter*^30^. Thus, the lack of methanogens in the final prediction models is not surprising. At low abundance, methanogens, fungi, and protozoa might have little predicting power for CH_4_ emissions.

Both the ANIM-B and DMI-B ML models included the genus *Oribacterium* and one unclassified genus of the order *Gastranaerophilales* (the Uncl_Family of the order *Gastranaerophilales*) with a negative and a positive coefficient, respectively. Although the function of this unclassified genus is not known, its large coefficients in both prediction models suggest a high predicting power. Interestingly, *Gastranaerophilales* has been shown to be negatively associated with milk yield in dairy cattle^31^ and negatively associated with growth in lambs^32^. Regarding the genus *Oribacterium*, it had a negative coefficient in both the final ANIM-B and the DMI-B models. This aligns with *Oribacterium* being a potential H_2_ sink in the rumen of sheep^33^ and its negative association with CH_4_ emissions documented in dairy cows^34^. Future research is warranted to explore and verify if *Oribacterium* has a quantitative relationship with rumen CH_4_ production and can be used as a predictor variable or biomarker of CH_4_ emissions.

In addition to the two genera shared with the DMI-B SCAD model, the ANIM-B glmmLasso model included another eight genera, five of which were unclassified, and their functions remain unknown. Among these genera, *Moryella*, *Prevotella*_7, and *Syntrophococcus* are known genera of bacteria, but their association or correlation with CH_4_ emissions in ruminants has not been reported in the literature. *Prevotella_*7 is a newly established genus^35^, consisting of *P. multiformis*, *P. multisaccharivorax*, and uncultured bacteria from the oral cavities and the rumen (https://www.arb-silva.de/). Studies have shown that both *P. multiformis* and *P. multisaccharivorax* were enriched by concentrate-based diets correlated with decreased CH_4_ emissions^36,37^. In buffalos, a higher relative sequence abundance of *Prevotella* (closely related to *Prevotella_*7) was associated with low CH_4_ emissions^38^. *Prevotella* is considered to be hydrogen-consuming^39^, making the inclusion of *Prevotella*_7 as a predictor variable with a negative coefficient not surprising. As for *Moryella* and *Syntrophococcus*, the literature has no information about their relationship with CH_4_ emissions, except for one in vitro study that showed a positive correlation between *Syntrophococcus* and maximum CH_4_ production, but a negative correlation with the appearance of peak CH_4_ production^40^. Future quantitative studies are needed to verify the qualitative relationship between these two genera and CH_4_ emissions.

The DMI-B SCAD model did not include BW as a predictor variable, but the inclusion of bacterial predictor variables improved the prediction accuracy. This suggests that these bacterial predictor variables might have reduced the variation in CH_4_ prediction introduced by BW. In addition to the two unknown genera discussed earlier, the final DMI-B SCAD model included three known genera (*Pseudoramibacter*, *Megasphaera*, and *Selenomonas*), which were not included in the final ANIM-B glmmLasso model. While the literature has no information about any potential association between *Pseudoramibacter* and CH_4_ emissions, *Pseudoramibacter* was reported to be positively correlated with feed efficiency in sheep^41^. Similarly, it remains unknown if *Megasphaera* or *Selenomonas* has any association or correlation with CH_4_ emissions in the absence of anti-methane inhibitors. However, one in vitro study has shown that calcium salts of long-chain fatty acids^42^, tucuma oil^43^, and ginkgo extract^44^ decreased CH_4_ emissions while increasing propionate and the abundance of *Megasphaera* and *Selenomonas*. Encapsulated nitrate has also been found to reduce CH_4_ emissions while increasing *Selenomonas* in steers^45^. Additionally, *Megasphaera* was more abundant in low than in high CH_4_-yield sheep^46^. Thus, the negative coefficients of both *Megasphaera* and *Selenomonas* in the DMI-B SCAD model align with their negative association with CH_4_ emissions and their ability to consume hydrogen and produce propionate^33,39,47,48^. It should be noted that many of these correlations were observed in ruminants that were subjected to dietary interventions using anti-methane inhibitors, which could directly or indirectly affect these genera, skewing their relationships with CH_4_ production. Future research is warranted to determine their relationship with CH_4_ emissions in the absence of anti-methane inhibitors and their predicting power of CH_4_ emissions.

### Potential limitations of the sheep dataset and the selected models

The dataset used in developing the prediction models was from one study using a relatively small number of sheep that were fed the same diet^13^. A larger database from multiple studies using different diets would improve prediction models and enhance their applicability across a broad range of feeding systems. However, currently, no such a database exists. A few studies in the literature have reported rumen microbiota data together with CH_4_ emission data, and we attempted to combine these data into a larger dataset. However, most of the studies used anti-methane inhibitors, likely skewing the microbiota data. Additionally, different studies sequenced different regions of the 16S rRNA or 18S rRNA gene, making it impossible to combine the sequence data for microbiota analysis. To facilitate the combination of sequence data from multiple studies, a standardized metataxonomic protocol should be adopted.

Metataxonomic analysis of the rumen microbiota incurs additional cost, which may hinder the adoption and application in the livestock industry, but it is relatively small and justifiable for several reasons. First, amplicon sequences are routinely generated and analyzed in numerous studies to understand the microbiological underpinning of treatment effects or group differences, making sequencing cost a part of the study cost. Second, the cost of amplicon sequencing has been steadily decreasing, and with the advent of new sequencing technologies (e.g., the Illumina NovaSeq 6000), it is expected to further decrease. Third, accurate and precise CH_4_ emission prediction is becoming increasingly important for precision livestock farming, which is an essential approach to increase feed efficiency while reducing CH_4_ emissions for a sustainable industry of ruminants. Improved prediction of CH_4_ emissions can assist researchers and farmers in improving the overall feed efficiency and sustainability of livestock production.

## Conclusion

Using machine learning methods, we successfully developed animal-based and dry matter intake-based CH_4_ prediction models that include both animal data and microbial genera as predictor variables. The inclusion of microbial predictor variables improved CH_4_ emission prediction in terms of error, bias, and accuracy compared to the conventional models that solely contain animal-related data, particularly for predicting CH_4_ yield (g CH_4_/kg of DMI). Notably, the coefficients (positive or negative) of some of the predictor variables from known bacterial genera (e.g., *Oribacterium*, *Prevotella_*7, *Megasphaera*, and *Selenomonas*) align well with their known associations or correlations (positive or negative) with CH_4_ emissions and/or with their known functions in the rumen ecosystem, such as hydrogen consumption. However, the role and association of other predictor variables of known genera (e.g., *Moryella*, *Pseudoramibacter*, and *Syntrophococcus*), as well as the unclassified genera, with CH_4_ emissions remain unknown.

While further improvements in CH_4_ prediction models would benefit from extensive databases encompassing rumen bacteria, archaea, fungi, and protozoa, along with animal-related data from multiple studies, this study demonstrated the potential and feasibility to include rumen microbes as predictor variables to improve CH_4_ predictions from sheep, likely from cattle also. A standardized approach to generate and analyze rumen microbial data is imperative to facilitate the compilation of multiple datasets from numerous studies. Such efforts will contribute to a better understanding of the rumen microbiota's impact on CH_4_ emissions and improved prediction models for sustainable agricultural practices.

### Supplementary Information

Supplementary Figures.

Supplementary Table 1.

Supplementary Table 2.

## Data Availability

The sequence and animal data used in this study were published by Kittelmann, et al.^13^ and can be downloaded from the EMBL database (accession numbers: ERP003779 for Bacteria, ERP003773 for Archaea, ERP003772 for ciliate protozoa, and ERP003764 for anaerobic fungi). The R codes used in this study are available at https://github.com/yu2269/CH4_prediction_model.

## Abbreviations

CH_4_: Methane
ANIM-B: Animal-based
A:P ratio: Acetate:propionate ratio
ITS1: Internal transcribed spacer 1
ASV: Amplicon sequence variant
VIF: Variance inflation factor
Uncl: Uncultured
BW: Body weight
DMI: Dry matter intake
DMI-B: Dry matter-based
ML: Machine learning
RF: Random forest
RF-B: Random forest combined with boosting
LASSO: Least absolute shrinkage and selection operator
glmmLasso: Generalized linear mixed with LASSO
SCAD: Smoothly clipped absolute deviation
AIC: Akaike information criterion
BIC: Bayesian information criterion
SE: Standard errors
SIS: Sure independence screening
MSPE: Mean square prediction error
RMSPE: Root mean square prediction error
MAE: Mean absolute errors
CCC: Concordance correlation coefficient
