# Supplementary material for: Methane prediction equations including genera of rumen bacteria as predictor variables improve prediction accuracy
Source: Sci Rep. 2023 Dec 2;13:21305. doi: 10.1038/s41598-023-48449-y (PMC10693554; doi:10.1038/s41598-023-48449-y)
Supplement: Supplementary file 1 — Supplementary Figures. [file 41598_2023_48449_MOESM1_ESM.pptx]

## Slide 1
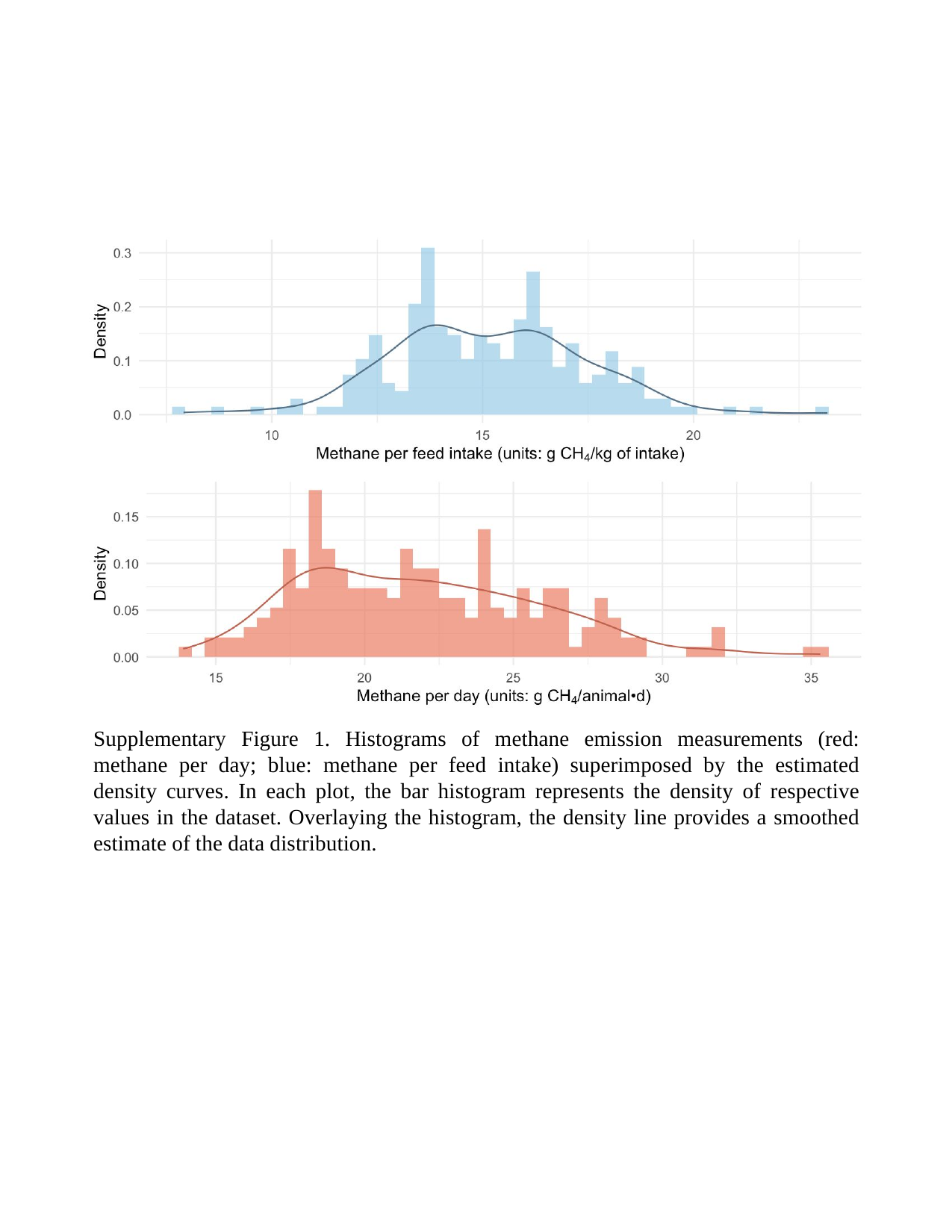

Supplementary Figure 1. Histograms of methane emission measurements (red: methane per day; blue: methane per feed intake) superimposed by the estimated density curves. In each plot, the bar histogram represents the density of respective values in the dataset. Overlaying the histogram, the density line provides a smoothed estimate of the data distribution.

## Slide 2
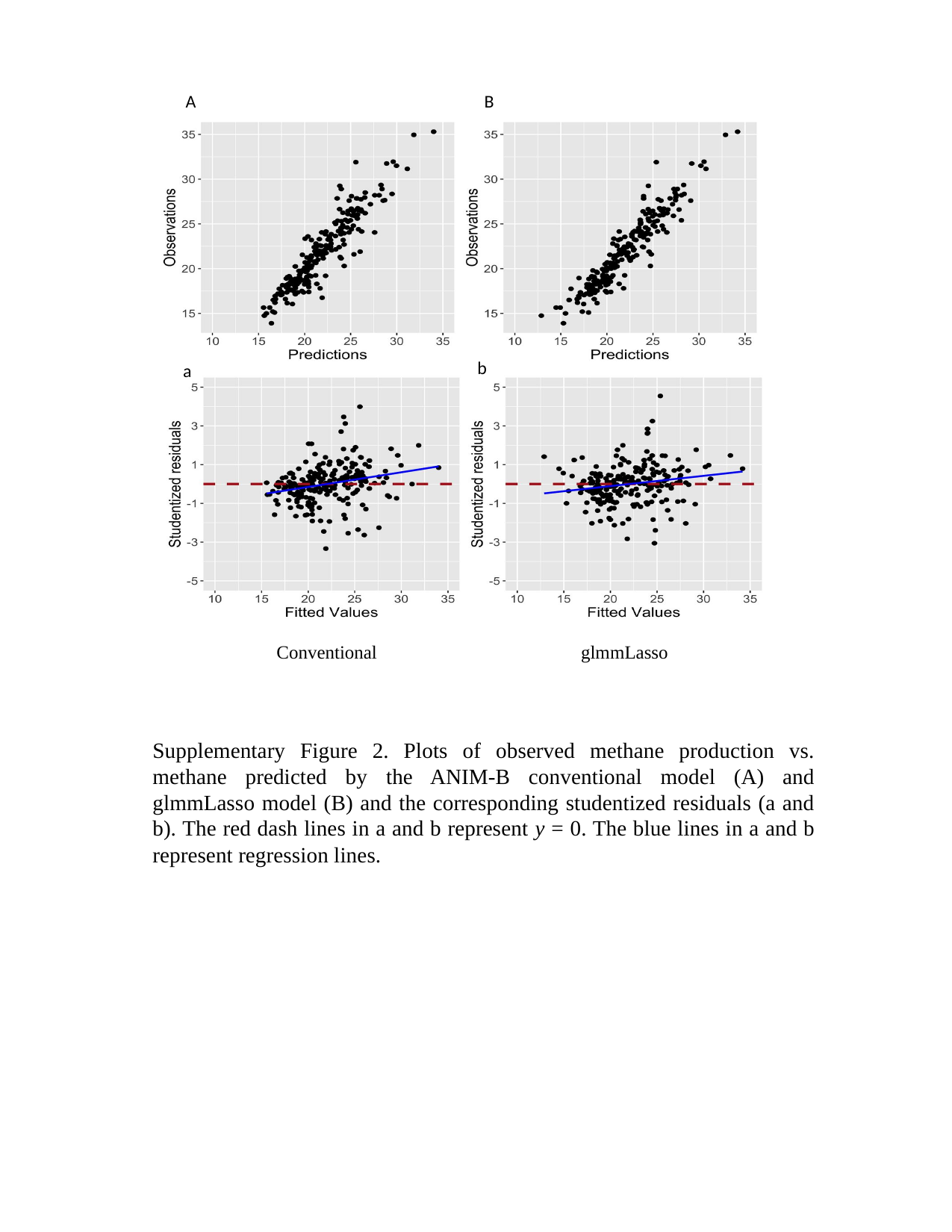

A
B
a
Conventional
glmmLasso
b
Supplementary Figure 2. Plots of observed methane production vs. methane predicted by the ANIM-B conventional model (A) and glmmLasso model (B) and the corresponding studentized residuals (a and b). The red dash lines in a and b represent y = 0. The blue lines in a and b represent regression lines.

## Slide 3
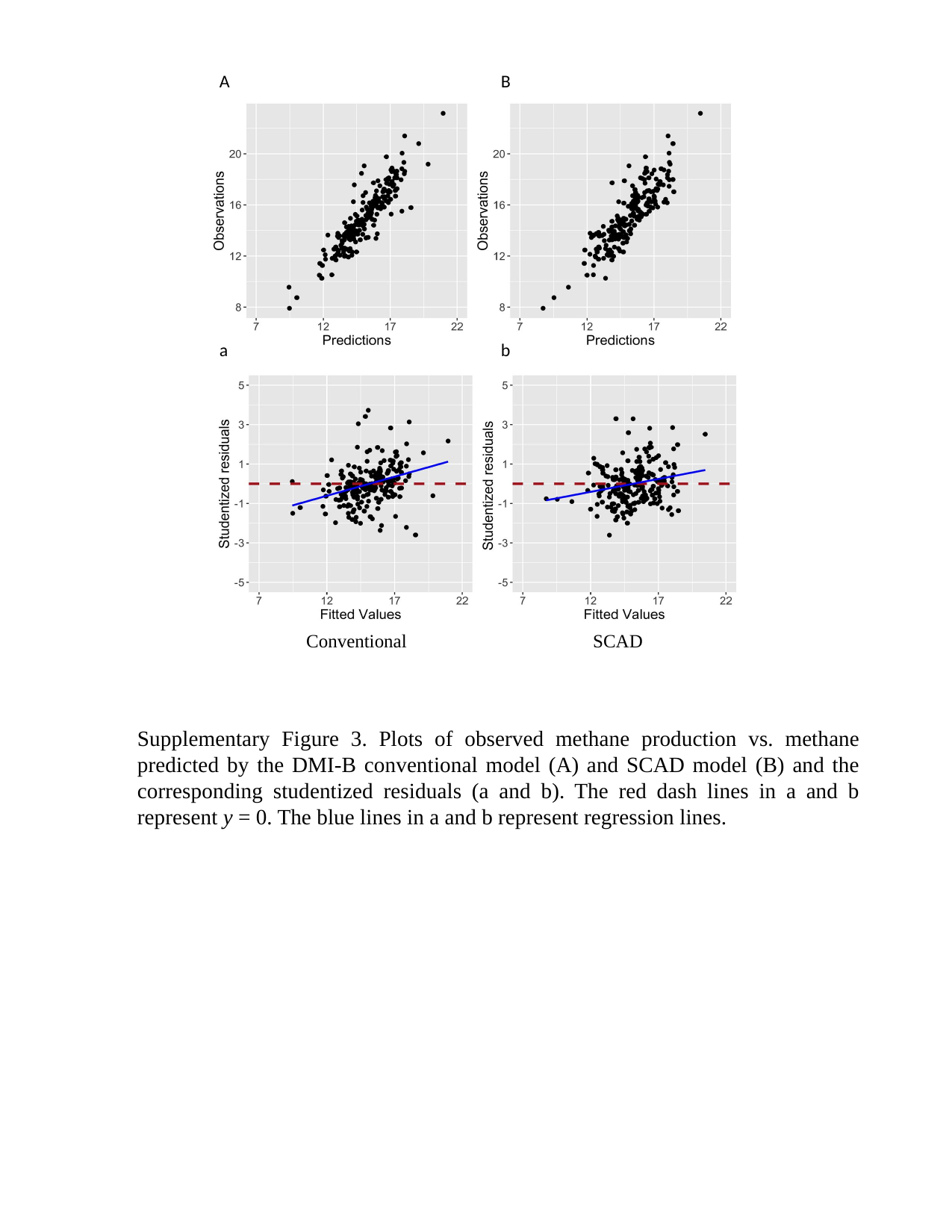

A
B
a
b
Conventional
SCAD
Supplementary Figure 3. Plots of observed methane production vs. methane predicted by the DMI-B conventional model (A) and SCAD model (B) and the corresponding studentized residuals (a and b). The red dash lines in a and b represent y = 0. The blue lines in a and b represent regression lines.
