# Supplementary material for: Methane prediction equations including genera of rumen bacteria as predictor variables improve prediction accuracy
Source: Sci Rep. 2023 Dec 2;13:21305. doi: 10.1038/s41598-023-48449-y (PMC10693554; doi:10.1038/s41598-023-48449-y)
Supplement: Supplementary file 2 — Supplementary Table 1. [file 41598_2023_48449_MOESM2_ESM.docx]

**Supplementary Table 1.** Summary statistics of the animal variables (except sex and breed) and microbes variables included in final models

|  |  | **Mean** | **Median** | **Min** | **Max** | **SD*** | **1Q*** | **3Q*** |
| --- | --- | --- | --- | --- | --- | --- | --- | --- |
| **Animal variables** | DMI (kg/d) | 1.45 | 1.44 | 0.89 | 2.12 | 0.18 | 1.33 | 1.57 |
|  | BW (kg) | 45.5 | 44.5 | 29 | 64 | 6.37 | 40.6 | 49.7 |
|  | Acetate : propionate ratio | 4.15 | 4.1 | 2.9 | 5.76 | 0.6 | 3.71 | 4.48 |
|  | CH_4_ (g/d) | 21.95 | 21.52 | 13.9 | 35.3 | 4.06 | 18.61 | 24.6 |
|  | CH_4_/DMI (g/kg) | 15.14 | 15.09 | 7.91 | 23.17 | 2.3 | 13.59 | 16.6 |
| **Microbe variables^**^** | Uncl_Family of the order *Gastranaerophilales* | 0.00589 | 0.00389 | 0 | 0.0493 | 0.00702 | 0.00135 | 0.00779 |
|  | Genus *Pseudoramibacter* | 0.00028 | 0 | 0 | 0.00584 | 0.00069 | 0 | 0 |
|  | Genus *Megasphaera* | 0.0014 | 0 | 0 | 0.03 | 0.00373 | 0 | 0.00089 |
|  | Genus *Selenomonas* | 0.00411 | 0.00212 | 0 | 0.04358 | 0.0062 | 0 | 0.00531 |
|  | Genus *Oribacterium* | 0.00411 | 0.00133 | 0 | 0.03995 | 0.00619 | 0 | 0.00703 |
|  | Uncl_Genus of *Oscillospiraceae* | 0.00162 | 0 | 0 | 0.02062 | 0.00311 | 0 | 0.00235 |
|  | Uncl_Genus of the order *Clostridia* | 0.0122 | 0.01051 | 0.00084 | 0.03974 | 0.00706 | 0.00724 | 0.01525 |
|  | Uncl_Family of the order *Gastranaerophilales* | 0.00589 | 0.00389 | 0 | 0.0493 | 0.00702 | 0.00135 | 0.00779 |
|  | Genus *Moryella* | 0.00048 | 0 | 0 | 0.01289 | 0.00157 | 0 | 0 |
|  | Uncl_Family of the order *RF39* | 0.00799 | 0.00677 | 0 | 0.03786 | 0.00577 | 0.00424 | 0.01074 |
|  | Genus *Prevotella 7* | 0.00065 | 0 | 0 | 0.07461 | 0.00589 | 0 | 0 |
|  | Uncl_Genus of *Marinifilaceae* | 0.0005 | 0 | 0 | 0.00819 | 0.00125 | 0 | 0 |
|  | Genus *Syntrophococcus* | 0.00195 | 0 | 0 | 0.02013 | 0.00386 | 0 | 0.00256 |
|  | Uncl_Family of *Oscillospirales* | 0.00344 | 0.00203 | 0 | 0.02068 | 0.00402 | 0 | 0.00558 |

***^*^***: SD: Standard Deviation. 1Q: First Quantile. 3Q: Third Quantile. Total number of data n = 218.

***^**^***: Log-transformed relative sequence abundance.
